# Association between Food Insecurity, Socioeconomic Status of the Household Head, and Hypertension and Diabetes in Maputo City

**DOI:** 10.5334/aogh.4569

**Published:** 2024-12-10

**Authors:** Elias M. A. Militao, Olalekan A. Uthman, Elsa M. Salvador, Stig Vinberg, Gloria Macassa

**Affiliations:** 1 Department of Health Sciences, Faculty of Humanities, Mid Sweden University, Holmgatan 10, 851 70 Sundsvall, Sweden; 2 Department of Social Work, Criminology and Public Health Sciences, Faculty of Occupational and Health Sciences, University of Gävle, Kungsbacksvägen 47, 80176 Gävle, Sweden; 3 Department of Biological Sciences, Faculty of Sciences, Eduardo Mondlane University, 3453 Julius Nyerere Avenue, Maputo 257, Mozambique; 4 Warwick Centre for Global Health, Division of Health Sciences, Warwick Medical School, University of Warwick, Coventry CV4 7AL, UK; 5 Department of Global Health, Division of Epidemiology and Biostatistics, Faculty of Health Sciences, Stellenbosch University, Francie van Zijl Drive, Cape Town 7505, South Africa; 6 Department of Health Sciences, Faculty of Humanities, Mid Sweden University, Kunskapens väg 8, SE-831 25 Östersund, Sweden; 7 EPI Unit–Instituto de Saúde Pública, Universidade do Porto, Rua das Taipas 135, 4050-600 Porto, Portugal

**Keywords:** food insecurity, socioeconomic status, physical health outcomes, hypertension and diabetes, heads of households

## Abstract

*Background:* Metabolic diseases such as hypertension and diabetes are increasingly recognized as not just medical issues, but as complex conditions influenced by various factors.

*Objectives:* This study aimed to explore the association between food insecurity (FI) and hypertension and diabetes and how socioeconomic status influences this relationship.

*Methods:* Based on a cross‑sectional study of 1,820 participants conducted in Maputo City, FI was measured using a modified version of the US Department of Agriculture scale; metabolic diseases were assessed using self‑reports of the actual diagnoses, and data were analyzed through multinomial regression and interaction terms.

*Results:* The findings revealed significant links between FI, socioeconomic status, hypertension and diabetes. Socioeconomic status had a clear influence on the association between FI and hypertension but showed a nuanced influence on diabetes. Specifically, regarding diabetes, the heads of households with a higher socioeconomic position were more likely to have this health condition than their counterparts with a lower socioeconomic position.

*Conclusions:* The study underscores the complex interplay between FI and socioeconomic status in influencing the risk of metabolic diseases. Addressing FI and improving socioeconomic status may be crucial steps in mitigating the risk of hypertension and diabetes among vulnerable populations, emphasizing the importance of a holistic approach to health promotion and disease prevention.

## Introduction

We live in a rapidly changing environment where high‑income countries (HICs) and low‑ and middle‑income countries (LMICs) are increasingly facing similar health issues, especially because of demographic aging, rapid urbanization, and globalization of unhealthy lifestyles. Today, non‑communicable diseases (NCDs) have overtaken infectious diseases as the world’s leading causes of mortality and morbidity [1].

The World Health Organization [2] has reported that NCDs kill 41 million people (equivalent to 74% of all deaths globally) every year, and more than three quarters of these deaths occur in LMICs. These conditions are often associated with older age groups, but estimates indicate that 17 million NCD deaths occur before the age of 70 years. Of these premature deaths, about 86% occur in LMICs. Overall, each year cardiovascular diseases (CVDs) account for most deaths (17.9 million), followed by cancers (9.3 million), chronic respiratory diseases (4.1 million), and diabetes (2 million) [2].

Hypertension is one of the key risk factors for CVDs. It already affects 1.3 billion people globally, leading to heart attacks and strokes [1]. Estimates indicate that hypertension kills 9.4 million people every year, though it can be prevented. In fact, prevention is far less costly, and far safer for patients, than medical interventions (e.g., cardiac bypass surgery and dialysis) that are needed when hypertension goes untreated. Hypertension and other risk factors such as diabetes often co‑occur. Hypertension is a silent killer, which causes hardly any symptoms in the early stages; hence, increasing public awareness is key to early detection. To this end, countries need systems and services in place to promote universal health coverage and support healthy lifestyles (e.g., healthy eating, avoiding alcohol and tobacco, and getting regular exercise). In parallel, access to quality medicines is also necessary, particularly at the primary care level [1].

Similarly, diabetes, a chronic metabolic disease characterized by elevated levels of blood sugar, can lead to serious damage to the heart, blood vessels, eyes, kidneys, and nerves. The most common diabetes type is type 2 diabetes (accounting for about 96%), which is usually found in adults and occurs when the body becomes resistant to insulin or does not produce enough insulin. Globally, the prevalence of type 2 diabetes has been progressively increasing, and it is estimated that about 529 million people (6.1% of the global population) have diabetes, with projections of 1.31 billion people having the disease by 2050 [3]. In 2020, the Lancet Commission on Diabetes highlighted the unequal burden of the disease, reporting that 80% of diabetes cases occur in LMICs. Preventing and controlling type 2 diabetes remains an ongoing challenge. To this end, it is critical to better understand the inequalities in risk factors across populations to inform policies and strategies to curtail the burden of NCDs in the context of multiple and complex drivers [3].

In Mozambique, research evidence indicates a prevalence of hypertension of 38.9% [4] and of type 2 diabetes of 7.4% [5]. A study by Jessen et al. suggests that both the distribution of cardiovascular risk factors and the awareness of these factors depend on the socioeconomic and demographic characteristics of the population. Likewise, a study by Madede et al. reports that only 10% of the studied people with diabetes were aware of their condition and fewer than 50% were on medication. These figures highlight the severity of the current situation in Mozambique as the prevalence of these conditions has been progressively increasing over the last decade and there is a gap in human capital, funding and material resources to effectively respond to the country’s needs in relation to NCDs [6].

There is great interest in how social determinants of health impact cardiometabolic diseases. Also well documented is that one common thread among cardiometabolic diseases is diet sensitivity [7, 8]. Food insecurity (FI) is heavily associated with poor diet quality because households cannot afford nutritionally adequate and safe food, which is expensive in comparison with highly refined foods containing large amounts of sugar and sodium [9]. Research evidence suggests that FI has a complex and bidirectional relationship with cardiometabolic diseases: (a) it may increase the risk for poor control of cardiometabolic diseases through poor diet; and (b) cardiometabolic diseases may increase FI through diminished ability to work, in combination with high medical expenses [10]. A systematic study by Miguel et al. found that FI was directly associated with all measured cardiometabolic risk factors (e.g., hypertension, dyslipidemias, diabetes) after adjustment for sociodemographic, economic, and lifestyle factors. Therefore, the findings recognize that FI and cardiometabolic diseases establish a vicious cycle in which each condition is strengthened [11]. They also emphasize the need to address FI as an integral component of diet‑sensitive NCD prevention programs [8].

Moreover, research evidence has highlighted the effects of socioeconomic disparities on household FI and physical health outcomes. Indeed, inequalities in income, education and work contribute significantly to household poverty and widespread FI in LMICs [12] and are associated with increased risk of poor physical health [13]. Poverty and low income, for instance, can prevent vulnerable populations from accessing quality food [14], thus increasing their risk of developing metabolic diseases. Likewise, other competing expenses such as adequate housing, quality health care and quality education are largely out of reach for the poor [15]; and they equally contribute to household FI and negative physical health. Education can influence how individuals and households access and utilize food [16]. It can make well‑educated people more aware of their health conditions and more receptive to health services compared with their less educated counterparts [15]. Education is also a proxy for better employment opportunities [16], and is closely linked to social prestige and networks [15]. Conversely, unemployment and lack of decent work among household heads can propel their families into poverty and FI to the point of adopting unhealthy behaviours (e.g. eating unsafe food and engaging in transactional sex), and thus contribute to an increased risk of having adverse physical health outcomes [9].

Despite this trend, there is a lack of information on the effects of household FI and socioeconomic status (SES) on hypertension and type 2 diabetes in the general population in Mozambique and Maputo City in particular. In fact, most empirical studies have explored these factors separately and have failed to examine their interaction effects [10, 17]. The studies by Irving et al. [18] and Helmick et al. [19] from outside Africa found FI to be associated with hypertension and diabetes after adjusting for socioeconomic indicators and other factors. Altogether, these findings suggest that FI may play a more significant role in the development of chronic diseases, especially among vulnerable populations [10]. Therefore, this study aimed to investigate the relationship between household FI and hypertension and type 2 diabetes among household heads in Maputo City, and how the SES of household heads influences this relationship.

## Materials and Methods

### Study setting and sampling

This cross‑sectional study was performed in Maputo City, the capital of Mozambique, between November 2021 and June 2022. The city is divided into seven municipal districts [20], and is the largest urban agglomeration in Mozambique. The city has about 1,080,277 inhabitants and 235,750 households [21]. It has about 65% of underemployment [20], and about 79% of households are food‑insecure [22].

A full description of the survey is provided elsewhere [22]. The selection of households relied on a two‑stage design inspired by Mozambique’s National Institute of Statistics (INE). This was used by the Mozambique Technical Secretariat for Food Security and Nutrition (SETSAN) in their 2013 baseline study [23]. In the first stage, a total of 96 enumeration areas were randomly selected, and each area provided a maximum of 20 households. In the second stage, households located within each enumeration area were selected using a systematic random sampling strategy (to select every fifteenth household), and based on approximate proportional allocation, a total of 1,842 households in four municipal districts (Nlhamankulu, KaMaxaquene, KaMavota, and KaMubukwana) were selected. However, for the purposes of this study, 22 of these households (whose heads were suffering from both hypertension and diabetes) were excluded from the data analysis.

## Measures

The primary outcome of this study was the metabolic disease index, a composite measure developed to capture the presence of metabolic diseases through self‑reported hypertension and diabetes. Based on the index, individuals were categorized into groups representing those with hypertension only, those with diabetes only, and those with neither condition, the last of which served as the reference category for our regression analysis. Respondents reporting both hypertension and diabetes were not included owing to their small numbers, which could undermine the statistical validity and reliability of the results. The assessment of both hypertension and diabetes relied on self‑reporting by household heads of actual diagnoses performed at a hospital or health center or medical clinic, confirmed through medical history (e.g. medical prescription). Specifically, the heads of households were asked “Have you ever been diagnosed with hypertension or diabetes or any other chronic disease?,” with a “yes/no” response to each health condition. All yes responses were followed up by asking, “When did it occur?” (probable year of diagnosis), and if they were currently taking any medication or treatment. Only those cases (actual diagnosis of hypertension or diabetes) that could be verified through medical history were considered.

Food insecurity was assessed using eight items from the US Department of Agriculture Household Food Security Module to measure FI in the last 3 months, as previously described elsewhere [22]. Cronbach’s alpha was 0.87, and households were considered food secure if they scored ≤1, scores of 2 or 3 were considered mild FI, scores from 4 to 6 were considered moderate FI, and scores of 7 or 8 were considered severe FI. For the study purposes, the first two categories were merged into one category during multinomial analysis. The questionnaire with all measurement instruments was in Portuguese and piloted in Manhiça district, a region outside the study setting. Dietary diversity was assessed on the basis of the variety and consistency of food items consumed in the last 7 days, and three categories were considered (low, medium, and high) [22]. Employment status was divided into two categories: paid work (including household heads with formal employment) and unpaid work (including those with informal employment). Educational attainment was categorized into primary or secondary education, versus high school or university education. Income levels were grouped into low, middle, and high income. These variables were chosen for their potential impact on the metabolic disease index, which was measured through self‑reported medical history, acknowledging the limitations that come with self‑reported data.

### Statistical analyses

Multinomial logistic regression was used to assess the associations between independent variables and the likelihood of having hypertension or diabetes, compared with the base outcome of having neither condition. The model’s goodness‑of‑fit was evaluated using the likelihood ratio chi‑squared test (LR *χ*^2^) and pseudo‑R‑squared statistics. All independent variables included in the final model were treated as covariates. The final model was built using the stepwise method and, in addition to the covariates, the model included the interaction terms (FI × Income, FI × Work and FI × Education) and their main effects.

Interaction terms were created to assess the moderation effects of socioeconomic factors on the relationship between FI and metabolic diseases. These included interactions between FI and education level, income category, and work type. These interaction terms were tested for significance and included in the final model if *p* < 0.05. In addition to the regression analysis, interaction plots were generated to visualize the moderation effects of socioeconomic variables on the relationship between FI and the presence of metabolic diseases. These plots provide a graphical representation of the predicted probabilities of metabolic disease outcomes as a function of FI status across levels of education, income, and employment status.

All analyses were conducted using either Stata 18 for Windows or IBM SPSS Statistics version 29, and results were considered statistically significant at *p* < 0.05. The regression coefficients, standard errors, *p* values, and 95% confidence intervals (CIs) were reported for all variables included in the final model.

## Results

### Summary statistics

The summary characteristics of the included participants, as shown in Table 1 below, present detailed demographics and socioeconomic factors across different metabolic index categories in the sample of 1,820 individuals. The average age of participants was approximately 33 years across all groups, with a standard deviation of around 9 years, indicating a relatively young demographic profile. In terms of FI, there was a notable difference between the groups. Altogether, 74.4% of individuals without any metabolic diseases were food insecure, which figure significantly increased to 93.7% in the hypertension group and 88.7% in the diabetes group. Food security was present in 25.6% of the “none” (= no metabolic disease) group, decreasing markedly in the hypertension and diabetes groups.

**Table 1:** Characteristics of the studied sample, Maputo City Household Survey, 2022.

	NONE* (*N* = 1,385)	HYPERTENSION (*N* = 364)	DIABETES (*N* = 71)	TOTAL (*N* = 1,820)	*P* VALUE (*X*^2^)
Age of participant, years	32.9 (9.1)	33.8 (8.8)	33.1 (9.9)	33.1 (9.0)	0.197
Food insecurity					
Food secure	355 (25.6%)	23 (6.3%)	8 (11.3%)	386 (21.2%)	<0.001
Food insecure	1,030 (74.4%)	341 (93.7%)	63 (88.7%)	1,434 (78.8%)	
Municipal district					
KaMaxaquene	377 (27.2%)	64 (17.6%)	25 (35.2%)	466 (25.6%)	<0.001
KaMubukwana	331 (23.9%)	109 (29.9%)	18 (25.4%)	458 (25.2%)	
KaMavota	323 (23.3%)	111 (30.5%)	12 (16.9%)	446 (24.5%)	
Nlhamankulu	354 (25.6%)	80 (22.0%)	16 (22.5%)	450 (24.7%)	
Sex of participant					
Male	500 (36.1%)	132 (36.3%)	24 (33.8%)	656 (36.0%)	0.921
Female	885 (63.9%)	232 (63.7%)	47 (66.2%)	1,164 (64.0%)	
Head of household					
Male	1,018 (73.5%)	235 (64.6%)	50 (70.4%)	1,303 (71.6%)	0.003
Female	367 (26.5%)	129 (35.4%)	21 (29.6%)	517 (28.4%)	
Household size	4.7 (1.0)	4.8 (0.8)	4.8 (0.9)	4.7 (0.9)	0.056
Number of children	2.0 (0.8)	2.2 (0.7)	2.1 (0.9)	2.0 (0.8)	<0.001
Marital status					
Single/separated	434 (31.3%)	147 (40.4%)	27 (38.0%)	608 (33.4%)	0.003
Married/marital union	951 (68.7%)	217 (59.6%)	44 (62.0%)	1,212 (66.6%)	
Food diversity					
Low	618 (44.6%)	287 (78.8%)	49 (69.0%)	954 (52.4%)	<0.001
Medium	487 (35.2%)	59 (16.2%)	17 (23.9%)	563 (30.9%)	
High	280 (20.2%)	18 (4.9%)	5 (7.0%)	303 (16.6%)	
Work					
Unpaid work	637 (46.0%)	214 (58.8%)	42 (59.2%)	893 (49.1%)	<0.001
Paid work	748 (54.0%)	150 (41.2%)	29 (40.8)	927 (50.9%)	
Education					
Primary/secondary	519 (37.5%)	261 (71.7%)	44 (62.0%)	824 (45.3%)	<0.001
High school/university	866 (62.5%)	103 (28.3%)	27 (38.0%)	996 (54.7%)	
Income					
Low income	385 (27.8%)	241 (66.2%)	29 (40.8%)	655 (36.0%)	<0.001
Middle income	536 (38.7%)	80 (22.0%)	25 (35.2%)	641 (35.2%)	
High income	464 (33.5%)	43 (11.8%)	17 (23.9%)	524 (28.8%)	

*No metabolic disease.

The distribution of participants across municipal districts showed significant variation, with different proportions of participants with a metabolic disease in the KaMaxaquene, KaMubukwana, KaMavota, and Nlhamankulu districts, suggesting potential geographical disparities in metabolic health conditions. Gender distribution across the groups was relatively balanced, with a slight majority of female respondents. However, there were significant differences in the gender of the household head, with more male‑headed households in the hypertension and diabetes groups. The overall household size was around 4.7 across the groups, and the number of children per household showed a significant difference, with more children in households with hypertension and diabetes.

Marital status varied significantly across groups, with a lower proportion of single, separated, or divorced individuals than of married individuals or those in a marital union having metabolic health conditions. Food diversity also showed significant differences, lower diversity being more common in the hypertension and diabetes groups. The type of work engaged in by participants was significantly different across groups, with a higher percentage of unpaid work in the hypertension and diabetes groups. Education levels varied significantly across groups, the hypertension and diabetes groups having a higher percentage of individuals with only primary or secondary education. Similarly, income categories showed significant differences, with the hypertension and diabetes groups having a larger proportion of individuals in the low‑income category.

### Association between food insecurity and self‑reported hypertension and diabetes

The results of multinomial logistic regression revealed positive and significant associations between FI, specifically moderate and severe FI and hypertension and diabetes (see Table 2). The odds ratio for hypertension and diabetes among food‑insecure households were about 3.05 (confidence interval; CI: [2.14−4.32], *p* < 0.001) and 3.48 (CI: [1.90−6.35], *p* < 0.001), respectively. Furthermore, SES, particularly education among heads with hypertension (0.53; CI: [0.37−0.75], *p* < 0.001), and household income among heads with diabetes (2.27; CI: [1.39−3.67], *p* < 0.001), displayed significant associations. In addition, there was a positive association between FI and household income among heads with diabetes. The proportion of participants across the municipalities (with KaMaxaquene as reference group) revealed a significant association with diabetes (0.78; CI: [0.63−0.95], *p* = 0.012). Age showed a significant association with hypertension (1.19; CI: [1.02−1.39],* p* = 0.025). Food diversity revealed some negative association with diabetes (0.57; CI: [0.30−1.05], *p* = 0.07). Finally, the odds ratio for hypertension on the combined effects between FI and income was 1.62 (CI: [1.01−2.56], *p* < 0.042).

**Table 2:** Association between food insecurity and self‑reported hypertension and diabetes by socio‑demographic characteristics, Maputo City Household Survey, 2022.

METABOLIC INDEX^A^		B	STANDARD ERROR	WALD	DF	*P* VALUE	ODDS RATIO	[95% CI]	
Hypertension	Intercept	−2.667	0.475	31.563	1	<,001			
	Food insecurity	1.113	0.179	38.886	1	<0.001	3.045	2.146	4.320
	Food diversity	−0.111	0.185	0.360	1	0.548	0.895	0.622	1.287
	Income	−0.237	0.155	2.326	1	0.127	0.789	0.582	1.070
	Work	0.211	0.150	1.987	1	0.159	1.235	0.921	1.657
	Education	−0.641	0.179	12.807	1	<0.001	0.527	0.371	0.748
	Age of participant	0.175	0.078	5.002	1	0.025	1.192	1.022	1.389
	FI × Income	0.479	0.235	4.152	1	0.042	1.615	1.018	2.561
	FI × Work	−0.333	0.359	0.861	1	0.353	0.717	0.355	1.448
	FI × Education	0.276	0.442	0.388	1	0.533	1.317	0.554	3.135
	Municipal district	0.033	0.058	0.328	1	0.567	1.034	0.923	1.157
Diabetes	Intercept	−4.491	0.823	29.763	1	<0.001			
	Food insecurity	1.248	0.307	16.563	1	<0.001	3.482	1.909	6.351
	Food diversity	−0.564	0.312	3.276	1	0.070	0.569	0.309	1.048
	Income	0.817	0.247	10.973	1	<0.001	2.265	1.396	3.674
	Work	−0.149	0.271	0.303	1	0.582	0.862	0.507	1.465
	Education	−0.494	0.295	2.795	1	0.095	0.610	0.342	1.089
	Age of participant	−0.066	0.138	0.232	1	0.630	0.936	0.715	1.225
	FI × Income	−0.144	0.453	0.100	1	0.751	0.866	0.356	2.106
	FI × Work	0.123	0.628	0.039	1	0.844	1.131	0.330	3.878
	FI × Education	0.937	0.858	1.192	1	0.275	2.552	0.475	13.715
	Municipal district	−0.254	0.101	6.316	1	0.012	0.776	0.636	0.946

^A^ The reference category is: no metabolic disease.

### Influence of socioeconomic status on the association between food insecurity and hypertension and type 2 diabetes

**Figure 1:**
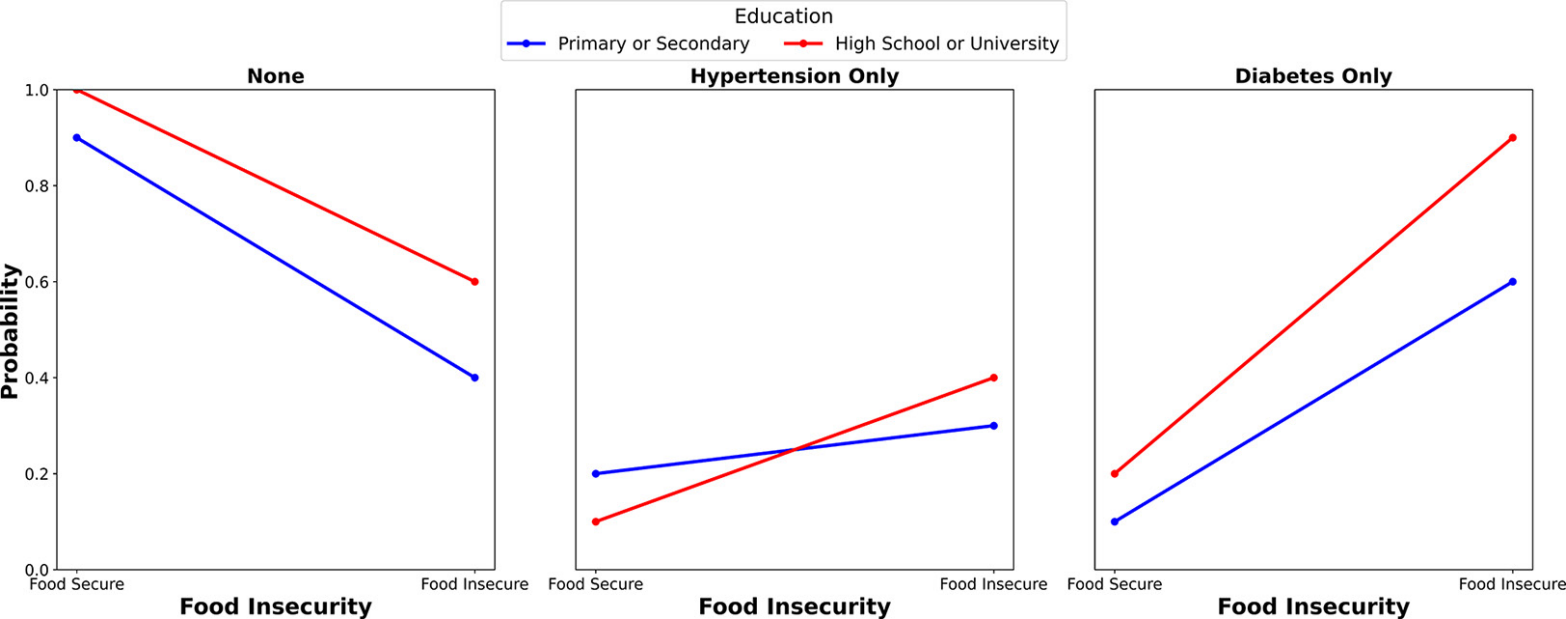
Adjusted prediction of the influence of educational attainment on the association between food insecurity (FI) and metabolic diseases.

Figure 1 gives a visual representation of the marginal probabilities of having no metabolic diseases, or having hypertension only, or diabetes only, across different levels of education and food security status. For each category of metabolic disease, two lines indicate the probability of individuals having or not having hypertension or diabetes by food security status (food secure versus food insecure) and educational attainment: primary or secondary education (blue) and high school or university education (pink). In the category labeled “none,” food‑secure individuals with a higher education displayed a much higher probability of having no metabolic diseases compared with their food‑insecure counterparts. A similar trend with a small magnitude was observed among individuals with a lower education. For the “hypertension only” category, the probability of having hypertension among food‑insecure individuals with a higher education was higher than that of their food‑secure counterparts, while those with a lower education displayed a similar trend with low magnitude. In the “diabetes only” category, a similar pattern was observed, with food‑insecure individuals with a higher education showing a higher probability of having diabetes compared with their counterparts with a lower education.

**Figure 2:**
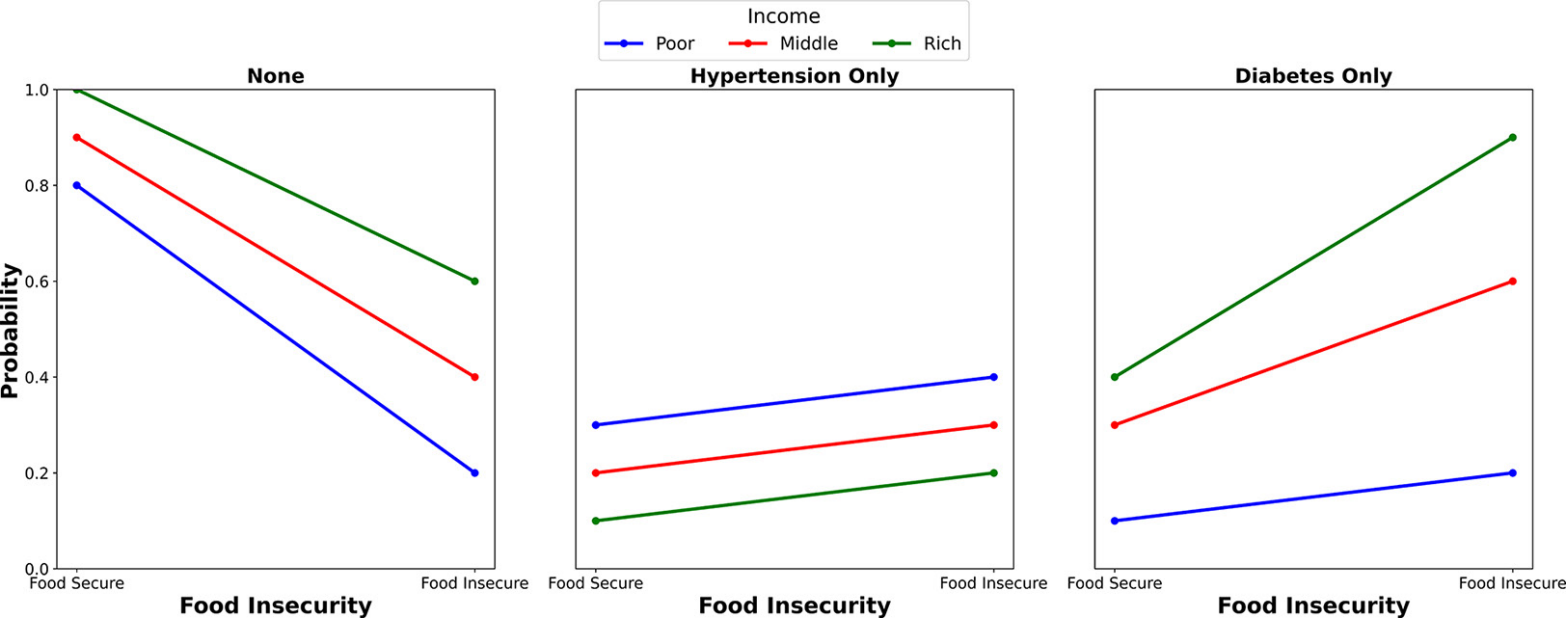
Adjusted prediction of the influence of income on the association between food insecurity (FI) and metabolic diseases.

Figure 2 depicts the marginal probabilities of individuals having no metabolic diseases, hypertension only, or diabetes only, stratified by food security status and income levels: low, middle, and high. Each metabolic condition category is illustrated by three lines, each representing the income level, and its relationship with food security status. In the category of no metabolic diseases (none), food‑secure individuals displayed a higher probability of having no metabolic diseases than their food‑insecure counterparts, irrespective of income levels. In the hypertension category, the trend across income levels was less pronounced, indicating a smaller gap in food security status, with the low‑income group more likely to suffer from FI and hypertension. In the diabetes category, the income gradient was sharply defined. Food‑insecure individuals with higher incomes showed a higher probability of having diabetes than their counterparts with lower incomes.

**Figure 3:**
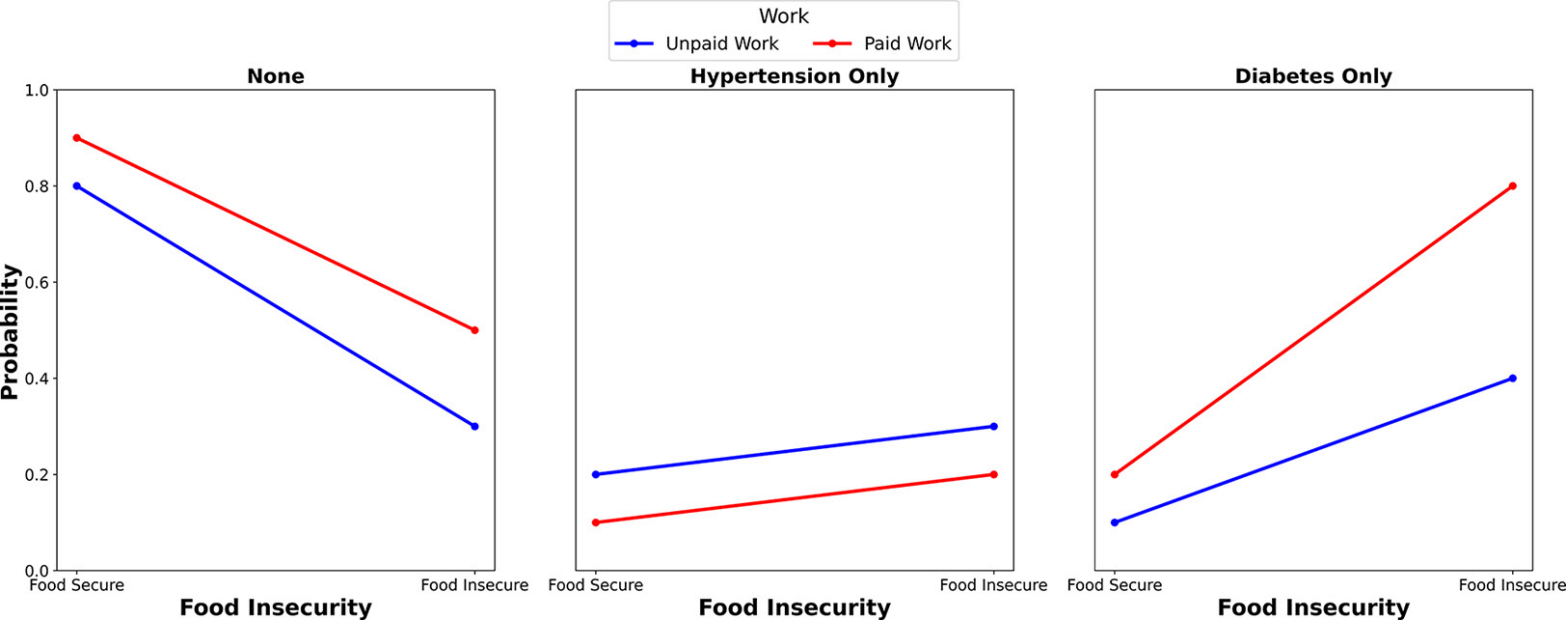
Adjusted prediction of the influence of employment status on the association between food insecurity (FI) and metabolic diseases.

Figure 3 illustrates the marginal probabilities of being classified with either no metabolic diseases, or hypertension only, or diabetes only, in relation to individuals’ work status and food security. The two lines in each metabolic condition category represent unpaid work (blue) and paid work (pink), each showing the probability of individuals having or not having hypertension or diabetes because of food security status. In the category of no metabolic disease (none), food‑secure individuals with paid work showed a much higher probability of having no metabolic diseases compared with their food‑insecure counterparts. A similar trend, but less pronounced, was observed among individuals with unpaid work. In the hypertension category, food‑insecure individuals with unpaid work displayed a relatively higher probability of having hypertension than their food‑secure counterparts. For paid work, the trend of having hypertension remained stable across food security status. Finally, in the diabetes category, food‑insecure individuals with paid work showed a higher probability of having diabetes than their counterparts with unpaid work.

## Discussion

This study sought to examine the relationship between household FI, the SES of the household head, and physical ill health (i.e., hypertension and type 2 diabetes) among household heads in Maputo City. The study showed that the combined effects of household FI and low SES of the household head were far more detrimental to people’s health in the studied sample than individual factors acting alone. A person’s health is influenced by various factors that may have synergistic or antagonistic effects on each other. These factors include age, sex, genetic factors, behaviours and life styles, social networks, environmental conditions, and health systems and services available to people [24].

Food insecurity as a social determinant of health is often linked to compromised food quality as observed in this study, and poor diet has been associated with higher risk of developing hypertension and diabetes [9]. Food insecurity in this study was more prevalent among households where the heads were suffering from hypertension and/or diabetes, suggesting the need for food security and nutritional interventions. There is a body of evidence indicating that a diet characterized by high intake of vegetables, fruit and complex carbohydrates and low intake of processed meat and refined carbohydrates may have a protective effect against hypertension, type 2 diabetes, CVDs, and other NCDs [25, 26]. Reporting from Namibia, Kazembe et al. found that a dietary pattern characterized by high intake of “starch–oil–sugars” and “meat–fish–dairy” was associated with hypertension, diabetes, and CVDs [27]. These products were consumed more by food‑secure households than by food‑insecure ones, which highlights the need for education on health and nutrition. Several studies have pointed out that food‑insecure households are more likely to buy cheap and unhealthy food, such as highly processed foods containing large amounts of sugar, sodium, and oils, and therefore have increased risk for NCDs [28, 29]. Findings from Madagascar and Cameroon also highlight the need for education on the nutritional value and benefits of consuming green leafy vegetables [30], as well as other neglected foods (e.g., soybean, Bambara bean and cowpea) for prevention of cardiometabolic diseases [31]. It has been found that although health concerns have been consistently reported as relevant when buying food, they were not translated into actual food choices because of cost [30].

Likewise, chronic stress and anxiety derived from household FI may contribute to insulin resistance and adiposity [32]. Research evidence, particularly from animal models, suggests that under food stress conditions, food intake is altered and there is a preference for high‑fat, high‑sugar foods [33, 34]. This activates the hypothalamic–pituitary–adrenal axis, releasing cortisol, which can alter metabolic processes and lead to increased visceral fat accumulation and storage, and amplify binge‑eating behaviors [34]. In turn, increased cortisol levels due to stress in humans are known to increase blood glucose and insulin resistance, which play critical roles in the development of type 2 diabetes [35]. Additionally, it has been shown in animal models that animals learn that high‑fat and high‑sugar foods reduce the stress response. Consequently, these animals seek the same food the next time stress is introduced, even with much lower stress stimuli [33]. The same behaviour applies to human beings.

Socioeconomic disparities are a core causal factor for health inequalities between and within societies [36] and are among the underlying causes of poverty and household FI, particularly in LMICs [12]. Each factor that constitutes SES (i.e., education, occupation, and income) has been reported to impact household food security [12] and physical health outcomes [37], as also observed in this study. As this study relied on self‑reporting, by household heads, of actual diagnoses performed at a hospital or medical clinic, our findings on diabetes may suggest that individuals at the top in education, income, and work are more aware of their health conditions than their counterparts in a lower status. Our findings also show the need for health services for the masses who cannot afford them. This corroborates with the findings by Madede et al. [5] who illustrated that only 10% of people with diabetes in Mozambique were aware of their condition. Furthermore, significant income disparities in food security status among diabetic individuals indicate the need for policy intervention to address financial inequities.

Education is the most basic SES component that shapes an individual’s career and financial potential, and can provide knowledge and skills that allow individuals with high educational attainment to have access to critical information and the resources needed to promote their health [38]. Furthermore, education can greatly influence food access and utilization among households and individuals [16]. Having high educational attainment is a proxy for better employment [16], social prestige and networks in modern societies [15]. In this study, education appeared to be a protective factor against hypertension, emphasizing the importance of resources and knowledge in health management.

Likewise, people from disadvantaged occupations are more likely to endure additional psychological distress, which may further exacerbate their physical health [37]. Unemployment and job insecurity can also have negative effects on health outcomes. Lower status occupations are more likely to expose workers to physical (labor injuries and exposure to toxic substances) and psychosocial risks (job strain and lack of control) [15] and, therefore, contribute to increased risk of cardiometabolic diseases. In similar ways, people with higher incomes have the means for better housing, schooling, health care, nutrition, and recreation [15, 38], leading to better health outcomes. However, in this study, multinomial results revealed a significant association between higher incomes and diabetes. This may reinforce the need for education on health and nutrition even among heads with a better SES to ensure they make appropriate food choices. Conversely, poverty and low income can hinder access to a quality diet and healthy eating [14], adequate housing and quality education [15]. Finally, daily financial hardships that vulnerable populations face bring down their sense of agency, control and self‑esteem, contributing to poor mental and, ultimately, physical health [39].

Individuals with a lower SES are more likely to suffer from FI [12] and ill health throughout their lives, as well as to develop illnesses earlier and spend more years with disability and die younger than those with a higher SES [38]. In this sense, individuals at the top have advantages in accessing resources, information, and circumstances that are more conducive to food security and better health outcomes [38, 40]. Therefore, coordinated and joint efforts from various actors and institutions, which effectively address each of these components as well as the underlying mechanisms through which they affect people’s physical health, are needed to close the gap between SES and household FI, especially among the most vulnerable groups.

It is noteworthy that, as observed in this study, inequalities in education, work, income, and other social factors where the risk for ill health increases with reductions in SES, may contribute heavily to the social gradient in health seen between and within societies [38, 41, 42]. This social gradient, where the poorest suffer most, is viewed as an injustice, and the adverse health outcomes (e.g., hypertension and type 2 diabetes) derived from it are health inequities that are avoidable and unfair [42]. Therefore, appropriate policies and interventions aimed to provide equal access to better education, and equal job opportunities should be implemented. Similarly, proportional interventions [38, 43] aimed to lift the most vulnerable groups out of poverty are equally necessary to close the gap in household FI and physical health outcomes. In short, this study supports a comprehensive approach to household FI and socioeconomic factors by shaping metabolic disease prevention and management. Future research should assess these relationships longitudinally and test interventions to mitigate disparities. At the same time, collaborations among health care providers, policymakers, and community organizations seem to be essential for strategy development and implementation of programs to alleviate household FI and improve physical health among vulnerable populations.

Apart from socioeconomic factors, in our study several other variables appeared to significantly mediate this complex relationship between household FI and physical health. For instance, geographic disparities in disease prevalence may suggest the influence of local resources and socioeconomic conditions. Likewise, marital status differences reveal potential impacts on physical health, with less social support possibly exacerbating ill health. The higher proportion of male‑headed households with metabolic diseases may indicate gender‑related disparities in health. Overall, age did not appear to play a significant role in influencing metabolic disease prevalence across groups (Supplementary Table 2), but it played a significant role in influencing hypertension. Besides the fact that the results were based on self‑reports of actual diagnoses, one of the main reasons that support this finding is that the studied population was fundamentally made up of relatively young people. Therefore, there is a need to consider all these key factors in the development of strategy policies and interventions to combat household FI and cardiometabolic diseases.

### Strengths and limitations

This is one of the few quantitative studies that provide recent empirical evidence on the relationship between the SES of the household head, and household FI and physical ill health (hypertension and type II diabetes) in Maputo City. The study had many participants (*n* = 1820) and used multinomial logistic regression (and a wide range of socioeconomic and demographic variables) for nuanced analysis. Also, the study examined the interaction effects between variables and generated interaction plots for clear visual representation of complex statistical data.

However, the findings need to be interpreted with caution and some limitations must be considered. A cross‑sectional design limits causality inference, and there is the potential for unmeasured confounding variables. In addition, reliance on self‑reported data may introduce response bias. There may be an underestimation of metabolic disease prevalence, especially from disadvantaged populations as this assessment relied on self‑report of actual diagnoses made at a hospital or medical clinic. Furthermore, assessing FI as a binary variable in the interaction effects analysis may have oversimplified its impact. Also, there were limitations in exploring the mechanisms underlying the observed associations between household FI, SES, hypertension, and type 2 diabetes.

## Conclusions

The study found a positive and statistically significant relationship between FI (moderate and severe FI) and hypertension and type 2 diabetes in Maputo City. Furthermore, the findings revealed that SES (particularly income and education) of the household head significantly influenced the relationship between household FI and physical ill health.

Addressing inequalities in household FI, along with SES, could therefore be a key strategy in promoting food security and healthy eating and better physical health in the studied population. In parallel, targeted policies and intervention programmes aimed to expand the coverage of key cardiometabolic diseases in terms of diagnosis and treatment and to make these health services more accessible to people are highly recommended. Similarly, targeted policies and intervention programs aimed to alleviate household FI (moderate and severe FI) and promote healthy eating, as well as promoting quality education and decent work and improving livelihoods among the most vulnerable groups are warranted.

## Data Availability

All the data supporting our findings have been presented in the manuscript. The datasets used are not publicly available owing to restrictions in the ethical approval for this study. However, de‑identified data can be made available by the corresponding author on reasonable request.

## Additional Files

The additional file for this article can be found as follows:

Supplementary File 1. Table 1. Model Fitting Information.

Supplementary File 2. Table 2. The main effects of the independent variables on metabolic index in the final model.
