# Supplementary material for: Association between Food Insecurity, Socioeconomic Status of the Household Head, and Hypertension and Diabetes in Maputo City
Source: Ann Glob Health. 2024 Dec 10;90(1):79. doi: 10.5334/aogh.4569 (PMC11639702; doi:10.5334/aogh.4569)
Supplement: Supplementary File 1. — Table 1. Model Fitting Information. [file agh-90-1-4569-s1.pdf]

Supplementary Material

S1 Table. Model Fitting Information

Model Fitting Information

Model	Model Fitting Criteria			Likelihood Ratio Tests		
	AIC	BIC	-2 Log Likelihood	Chi-Square	df	Sig.
Intercept Only	1429.284	1440.321	1425.284			
Final	1165.508	1286.917	1121.508	303.776	20	<,001

Goodness-of-Fit

	Chi-Square	Df	Sig.
Pearson	988.300	974	.368
Deviance	755.758	974	1.000

Pseudo R-Square

Cox and Snell	.154
Nagelkerke	.206
McFadden	.122
