# Supplementary material for: Association between Food Insecurity, Socioeconomic Status of the Household Head, and Hypertension and Diabetes in Maputo City
Source: Ann Glob Health. 2024 Dec 10;90(1):79. doi: 10.5334/aogh.4569 (PMC11639702; doi:10.5334/aogh.4569)
Supplement: Supplementary File 2. — Table 2. The main effects of the independent variables on metabolic index in the final model. [file agh-90-1-4569-s2.pdf]

Supplementary Material

S2 Table. The main effects of the independent variables on metabolic index in the final model

Likelihood Ratio Tests

	Model Fitting Criteria			Likelihood Ratio Tests		
Effect	AIC of Reduced Model	BIC of Reduced Model	-2 Log Likelihood of Reduced Model	Chi-Square	df	Sig.
Intercept	1216.793	1327.165	1176.793	58.621	2	<,001
Food Insecurity	1212.320	1322.693	1172.320	54.148	2	<,001
Food Diversity	1161.738	1272.110	1121.738	3.566	2	.168
Income	1173.146	1283.518	1133.146	14.974	2	<,001
Work	1160.830	1271.202	1120.830	2.658	2	.265
Education	1172.785	1283.157	1132.785	14.613	2	<,001
Age	1163.904	1274.277	1123.904	5.732	2	.057
FI * Income	1162.315	1272.687	1122.315	4.143	2	.126
FI * Work	1159.086	1269.458	1119.086	.914	2	.633
FI * Education	1159.820	1270.192	1119.820	1.648	2	.439
Municipal District	1165.628	1276.000	1125.628	7.456	2	.024
